# Using a stakeholder-engaged, iterative, and systematic approach to adapting collaborative decision skills training for implementation in VA psychosocial rehabilitation and recovery centers

**DOI:** 10.1186/s12913-022-08833-2

**Published:** 2022-12-17

**Authors:** Emily B. H. Treichler, Robert Mercado, David Oakes, Dimitri Perivoliotis, Yuliana Gallegos-Rodriguez, Elijah Sosa, Erin Cisneros, William D. Spaulding, Eric Granholm, Gregory A. Light, Borsika Rabin

**Affiliations:** 1 Desert Pacific Mental Illness Research, Education, and Clinical Center (MIRECC), VA San Diego, 3500 La Jolla Village Drive, San Diego, CA 92161 USA; 2 Department of Psychiatry, UC San Diego, 9500 Gillman Drive, La Jolla, CA 92037 USA; 3 Center of Recovery Education, VA San Diego, 3500 La Jolla Village Drive, San Diego, CA 92161 USA; 4 Department of Psychology, University of Nebraska-Lincoln, 238 Burnett Hall, Lincoln, NE 68588 USA; 5 Herbert Wertheim School of Public Health and Human Longevity Science, University of California San Diego, 9500 Gillman Drive, La Jolla, CA 92037 USA; 6 Clinical and Translational Research Center Dissemination and Implementation Science Center, UC San Diego Altman, UC San Diego, 9500 Gillman Drive, La Jolla, CA 92037 USA

**Keywords:** Adaptation, Stakeholder engagement, Implementation, VHA, Collaborative decision making, Serious mental illness

## Abstract

**Background:**

Adaptation of interventions is inevitable during translation to new populations or settings. Systematic approach to adaptation can ensure that fidelity to core functions of the intervention are preserved while optimizing implementation feasibility and effectiveness for the local context. In this study, we used an iterative, mixed methods, and stakeholder-engaged process to systematically adapt Collaborative Decision Skills Training for Veterans with psychosis currently participating in VA Psychosocial Rehabilitation and Recovery Centers.

**Methods:**

A modified approach to Intervention Mapping (IM-Adapt) guided the adaptation process. An Adaptation Resource Team of five Veterans, two VA clinicians, and four researchers was formed. The Adaptation Resource Team engaged in an iterative process of identifying and completing adaptations including individual qualitative interviews, group meetings, and post-meeting surveys. Qualitative interviews were analyzed using rapid matrix analysis. We used the modified, RE-AIM enriched expanded Framework for Reporting Adaptations and Modifications to Evidence-based interventions (FRAME) to document adaptations. Additional constructs included adaptation size and scope; implementation of planned adaptation (yes–no); rationale for non-implementation; and tailoring of adaptation for a specific population (e.g., Veterans).

**Results:**

Rapid matrix analysis of individual qualitative interviews resulted in 510 qualitative codes. Veterans and clinicians reported that the intervention was a generally good fit for VA Psychosocial Rehabilitation and Recovery Centers and for Veterans. Following group meetings to reach adaptation consensus, 158 adaptations were completed. Most commonly, adaptations added or extended a component; were small in size and scope; intended to improve the effectiveness of the intervention, and based on experience as a patient or working with patients. Few adaptations were targeted towards a specific group, including Veterans. Veteran and clinician stakeholders reported that these adaptations were important and would benefit Veterans, and that they felt heard and understood during the adaptation process.

**Conclusions:**

A stakeholder-engaged, iterative, and mixed methods approach was successful for adapting Collaborative Decision Skills Training for immediate clinical application to Veterans in a psychosocial rehabilitation center. The ongoing interactions among multiple stakeholders resulted in high quality, tailored adaptations which are likely to be generalizable to other populations or settings. We recommend the use of this stakeholder-engaged, iterative approach to guide adaptations.

## Contributions to the literature

- Mental health interventions are usually adapted to new populations or setting. Engaging stakeholders including patients, clinicians, and administrators in an iterative, mixed methods adaptation process increases quality and usefulness of the resulting adapted intervention.
- Interactions between stakeholders during adaptation who hold different positions in the same system has benefits and drawbacks. Earning trust and providing flexibility is key.
- For the intervention we adapted, Collaborative Decision Skills Training, most adaptations meant to improve effectiveness. Although many adaptations were “small” in terms of how much they changed the intervention’s content, the perceived impact among stakeholders was sometimes quite significant.

## Introduction

When mental health interventions developed in specialized settings are transferred to usual care settings for testing effectiveness, adaptations to the intervention are typically required [1, 2]. Adaptations are generally made to optimize the fit between the intervention and the specific context (i.e., setting, cultural group, diagnostic group, and care delivery agents) where the intervention is implemented [3]. These adjustments to the intervention are expected and essential to ensure proper implementation and sustained delivery of the intervention.

Adaptations often occur spontaneously when implementing interventions in the usual care setting [2, 4]. When such adaptations are made using a well-intentioned but ad hoc or reactive approach, it becomes increasingly likely that the changes will ultimately result in decreased fidelity and lessened effectiveness [5]. Additionally, spontaneous adaptations that are fidelity-consistent may be difficult to replicate by other clinicians or in other settings because they are usually not sufficiently documented [3–5]. It is therefore important to find strategies for systemically adapting interventions in usual care settings that allow for the optimization of the fit for the intervention in the specific usual care setting while maintaining its core functions. Furthermore, systematic documentation of these adaptations increases our ability to describe the adaptations and their impact, and replicate them in other settings, if they are useful and effective [2, 6].

Engagement of stakeholders in guiding systematic adaptations prior to implementation is a promising strategy to support optimization of the intervention for the local context and to ensure intervention relevance and fit [7, 8]. Mental health interventions have several potential stakeholders that might be included in the adaptation process, including patients, clinicians, administrators, and researchers. Meaningfully engaging all of these stakeholders is important because they represent important and distinct perspectives, values, and concerns [9, 10]. Furthermore, to acknowledge the complexity of bringing together multiple perspectives and making modifications to complex interventions, an iterative approach to intervention adaptation is desirable.

The Veterans Health Administration (VHA) in the United States prioritizes implementation of evidence-based mental health interventions [11] in a Veteran-oriented service model [12]. The VHA has nationally established Psychosocial Rehabilitation and Recovery Centers (PRRCs; [13, 14]) for Veterans with serious mental illness (SMI). PRRCs integrate evidence-based practice for Veterans with SMI with recovery-oriented services that conceptualize recovery as a holistic process centered around pursuit of personal meaning, connectedness, valued roles, empowerment, and improved functioning alongside symptom management [15]. In this context, recovery-oriented service models include collaborative decision-making, or meaningful engagement of Veterans in treatment decisions, given its focus on individualized and person-centered care, and on empowering Veterans in all aspects of their lives [16, 17]. However, current levels of Veteran involvement in treatment decisions are low [18], for a broad range of reasons at both the Veteran/clinician level and the institutional level [16].

Collaborative Decision Skills Training (CDST) is a promising intervention to improve knowledge, skills, comfort and confidence initiating and engaging in decision-making among people with SMI [19]. The aims of CDST are well-aligned with the goal of increasing Veteran voice in treatment, including treatment decision-making. CDST was originally piloted in a civilian service setting prior to expansion to VA. The VA service environment significantly differs from civilian contexts in institutional (e.g., size, mental health services offered, level of integration, payor process) and population characteristics (e.g., gender, age distribution, socioeconomic status, insurance status). Systematic adaptation is required as a prelude to larger scale implementation within VA. Therefore, we used an iterative, stakeholder-engaged approach to adapt CDST for use in Veterans with SMI in the context of a PRRC. This paper thus describes generalizable methods used to identify and complete intervention adaptations, the outcomes of our use of these methods, and reflects on lessons learned. Although this initial, pre-implementation adaptation did not include testing the adapted version of CDST, we did hypothesize that there would be evidence of successful adaptation via stakeholder feedback surveys.

## Methods

**Fig. 1:**
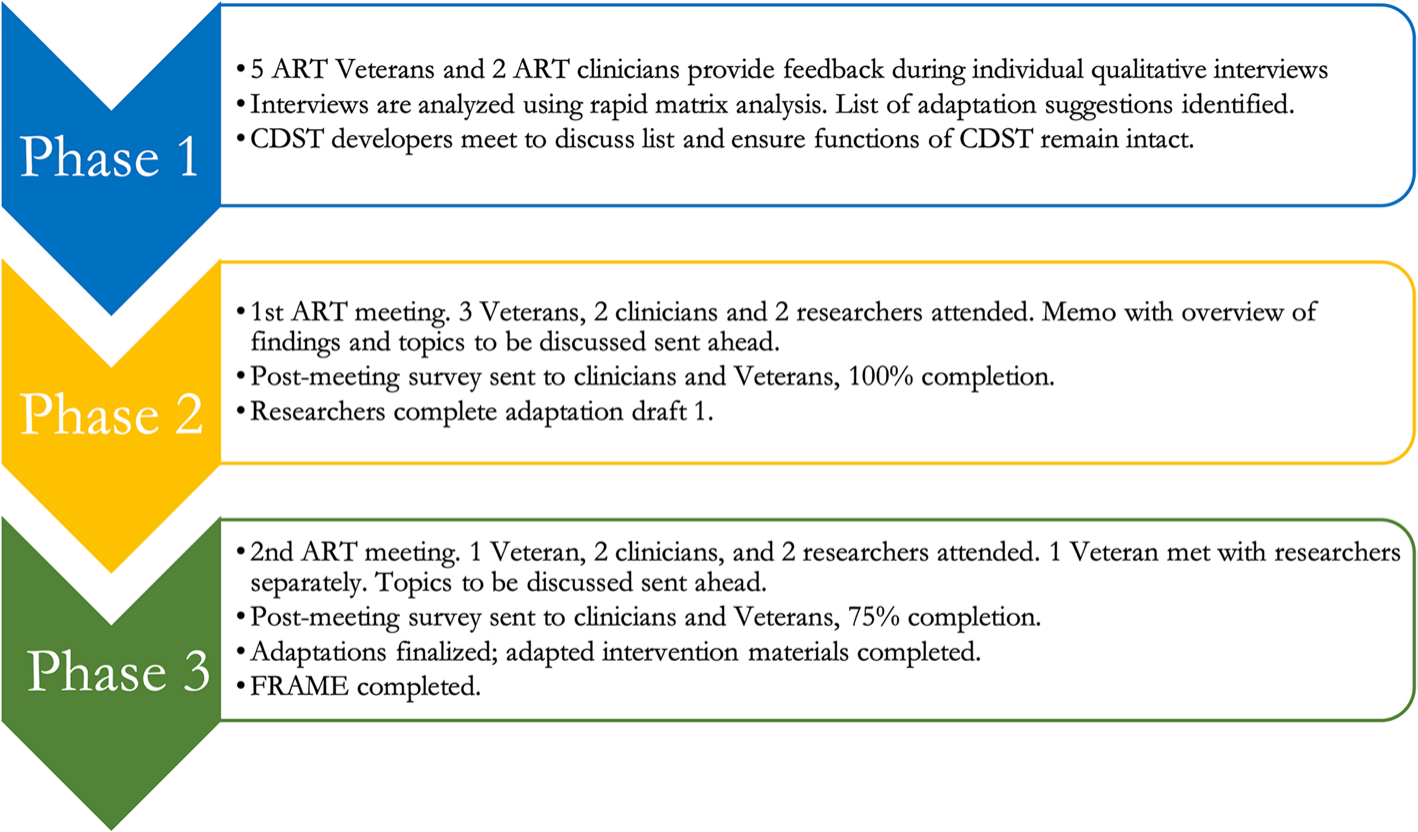
Study Design. Note: ART = Adaptation Resource Team. CDST = Collaborative Decision Skills Training. FRAME = Framework for Reporting Adaptations and Modifications

**Fig. 2:**
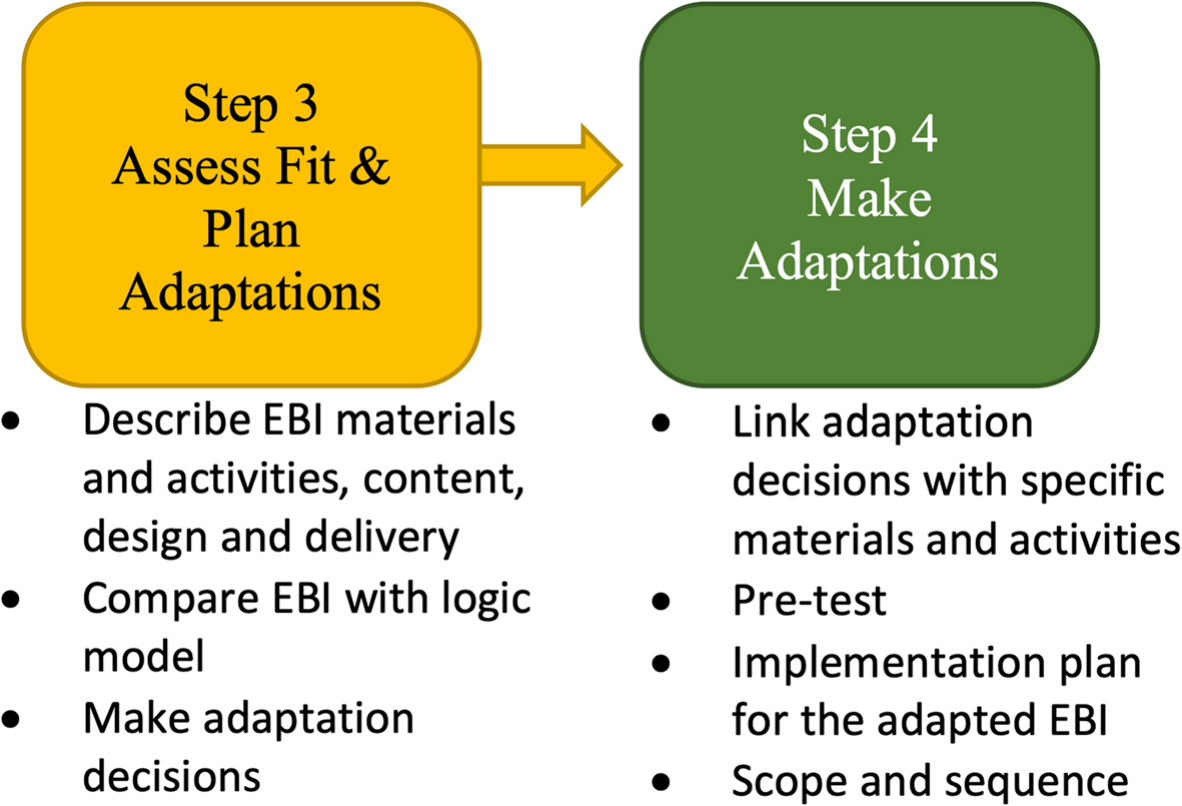
Steps of IM Adapt used in this study (adapted from Highfield et al., 2015)

Please see Fig. 1 for an overview of the study design. A mixed methods approach guided by the IM Adapt [20], a modified version of Intervention Mapping, and engagement from diverse stakeholders through an Adaptation Resource Team to systematically adapt CDST prior to implementation in the VA PRRC setting for Veterans with SMI (see Fig. 2). To systematically characterize and document these adaptations, we used the Reach, Effectiveness, Adoption, Implementation, and Maintenance (RE-AIM) enriched version of the expanded framework for reporting adaptations and modifications to evidence-based interventions (FRAME; [2, 6]). A protocol including the study described here was previously published [17]. We used the StaRI checklist [21] given that the StaRI includes both implementation and intervention elements.

### Study setting and target population

This study was conducted in a VA PRRC in a large city in Southern California. As described above, PRRCs are a national VA program for Veterans with SMI and provide comprehensive outpatient care using a recovery-oriented rehabilitation approach, including medication management, individual therapy, group therapy and skills training, supported education and employment, peer support, and chaplain services. Engagement in these services are coordinated by a recovery coach, who works individually with each Veteran to identify person-centered goals and tailor care to those goals. This PRRC includes an interdisciplinary treatment team and a large interdisciplinary training program. Although this PRRC mirrors other PRRCs nationally in many aspects given national mandates and best practices, it is unusual in that Veterans served in the program must have a psychosis diagnosis, while most other programs are more expansive in diagnostic inclusion criteria. Regardless, Veterans across programs meet functional definitions of SMI, meaning that they experience higher levels of illness severity, higher chronicity, and greater impact on functioning.

### Intervention

Collaborative Decision Skills Training (CDST) is an 8 session, group-based, skills training based on the collaborative decision-making model [16, 22] that incorporates skills related to goal-setting, assertiveness, problem-solving, and conflict resolution [19]. These skills are all discussed specifically in terms of their utility for improving patient engagement in treatment decision-making, satisfaction with treatment, and more broadly patient activation. Skills are set within a larger context of empowerment. Key forms and functions [23] for the CDST are summarized in Table 1. Clinicians providing CDST receive a manual that includes each session’s activities, handouts, and homework as well as guidance for session delivery, while participants receive a handbook that includes only the activities, handouts, and homework. Additionally, participants receive a laminated 4 × 6 overview card with basic information from skills taught in CDST that they can carry in a wallet or purse in they choose.

**Table 1:** CDST Functions and Forms

Core Functions (Standardized)	Forms (Tailored)
Empowerment-focused therapeutic approach that enhances overall empowerment, and specific feelings of empowerment related to participation in one’s own mental health care and recovery	Examples: • Psychoeducation about patient rights • Validation of desire to participate in treatment decision-making • Exploration of specific treatment preferences and goals • Treatment team handout and discussion of desired role on treatment team • Application of NOW, ASAP, and SCALIE to work towards pursuit of increased treatment participation or improved treatment satisfaction, among other possible goals
Evidence-based skills training strategies to improve ability to initiate and engage in collaborative decision-making and related skills when desired	Examples: • Role-plays • Worksheets • *Relevant examples/vignettes* • Application of discussed skills strategies to personal concerns and goals • In group discussion • In session practice of skills • Out of session practice of skills
Training in specific skills as they specifically apply to collaborative decision-making and related skills: treatment decision identification and goal setting; assertiveness; problem-solving; conflict resolution; using coping strategies to increase ability to engage in collaborative decision-making and related skills	Examples: • NOW • ASAP • SCALIE • *“Putting it all together” worksheet* • *Treatment decision checklist* • *Resolving disagreements with clinicians diagram* • *Coping skills practice*
Psychoeducation on relevant topics	Examples: • Provide full manual and overview cards • Discuss what collaborative decision-making is • Pros and cons of collaborative decision-making • *Treatment decision checklist* • Treatment team worksheet
Increase comfort and confidence related to participating in treatment decision-making	Examples: • ASAP • Discussion of past experiences with clinicians and how that may shape willingness, comfort, confidence to try collaborative decision-making with new clinicians • *Coping skills* • *Conflict and disagreement session and exercises*
Consider, validate, and support patients in identifying possible solutions for patient level, patient/clinician level, and patient/system level barriers to collaborative decision-making and other aspects of care	Examples: • Pros and cons of collaborative decision-making Discussion of past experiences with clinicians and how that may shape willingness, comfort, confidence to try collaborative decision-making with new clinicians • *Coping skills* • *Conflict and disagreement session and exercises* • Advocacy and system advocacy tools

*Italicized* forms were added or substantially adapted during the adaptation process described in this paper

### Adaptation resource team

The Adaptation Resource Team (ART) of diverse stakeholders was established to guide and provide perspective throughout the adaptation process to ensure that the VA-adapted CDST materials improved fit for the needs of Veterans with psychosis participating in VA PRRC services. Four groups of stakeholders were included in the team: Veterans with psychosis currently participating in VA PRRC services; VA mental health clinicians working in the PRRC; VA administrators involved in PRRC services; and researchers with relevant expertise. The ART included five Veterans; two clinical psychologists, both of whom also held administrative roles; and four researchers, including two CDST developers and expertise in SMI (two researchers), VA and Veterans (3), clinical training broadly (3), qualitative and mixed methods (3), and implementation science (2). All members of the ART were offered the opportunity to contribute to this paper and other research products associated with this project. This manuscript includes eight authors from the ART, two Veterans, two clinicians, and four researchers, who co-developed this manuscript. One Veteran ART member passed away following primary data collection. The other two Veterans elected not to participate in the development of this paper but continue to be involved in the research project.

### Adaptation procedures

The adaptation process followed steps 3 and 4 of IM Adapt [20] (Fig. 2). First, each Veteran and clinician/administrator ART member received the CDST materials, including the CDST participant manual, the CDST clinician manual, as well as a description of the of purpose of the in-development Service Delivery Manual. The Service Delivery Manual is a document that accompanies the clinician manual (CDST content) and describes CDST delivery, including population and setting considerations for effective group therapy delivery, approved ways to modify CDST delivery (e.g., number of sessions, length of sessions), and recommendations for dealing with common issues. Once ART members reviewed the materials, two ART research members (ET & RM) conducted individual semi-structured qualitative interviews assessing for relevance, anticipated impact, and ease of delivery, participation, and learning for CDST overall, and each major component of the materials. These data were collected during the COVID-19 pandemic, so the viability of delivering CDST fully virtually and the impact of virtual services on care was also queried.

*We used* primarily neo-positivist conceptualization for the semi-structured interviews and the other elements of the study [24]. At the same time, given that the ART included two CDST developers (ET and WS), and one of those developers was an interviewer (ET), we also integrated transparency and disclosure of positionality (i.e., that ET developed CDST) [24, 25]. Interviewers aimed to increase interviewees’ willingness to disclose negative feedback in two ways; the first, by explicitly stating at the beginning of each interview that all opinions were valuable, whether positive or negative, and that the purpose of the study was to benefit Veterans, not CDST. The second was to include questions that asked directly for negative feedback.

The interview transcripts were coded using rapid matrix analysis [26]. Themes were created a priori based on topics targeted in the interview guide, and a codebook was developed and completed through consensus meetings between three of the ART research members (ET, RM, and BR). The codes used included suggestions for adaptations, including whether the suggestion was to “keep,” “add,” “remove,” or “adapt,” an element of CDST. Coders (ET, RM) reviewed each other’s coding to ensure accuracy. BR acted as consensus expert for disagreements. Following completion, suggestions were compiled and separated into those in need of further discussion with ART from those that were easy to complete with minimal impact on overall intervention.

Following analysis, the two CDST developers (ET and WS) met to discuss the major suggestions to discuss ways to appropriately respond to suggestions in general and ensure that the functions of CDST would remain intact and forms would remain feasible [23]. Suggestions to be discussed were compiled into a short memo sent ahead of the first ART meeting. ART members were also offered the option to meet with the primary research team members (ET and/or RM) individually instead or in addition of the ART meeting if desired. The structure of the meeting was minimal in order to adapt to the priorities of the team members. In general, each suggestion was presented to the group and discussed until consensus formed about appropriate adaptation(s).

Veteran and clinician ART members were asked to complete a survey after the meeting which assessed the perceived importance, Veteran benefit, impact on ease of use and delivery for each major adaptation suggestion. It also assessed how heard and understood each ART member felt and used two open-ended questions to identify most important adaptations and any topics that should have been discussed but weren’t. The results of these surveys guided adaptation decisions, provided evidence of whether adaptations selected were successful (e.g., important to stakeholders, perceived to be likely to benefit Veterans, perceived to have implementation feasibility), and provided evidence of whether the stakeholder-engaged adaptation strategy itself was successful (i.e., whether stakeholders felt heard and understood).

Following the first ART meeting, research ART members (ET, RM, BR) met to resolve recommended changes that hadn’t yet reached consensus. Research ART members focused on resolving adaptations by integrating feedback from other ART members and evidence from implementation science to maximize feasibility and acceptability. A first draft of adaptations to the clinician manual, participant handbook, overview card and to the service delivery manual were then completed. A new card for coping skills was added. Each change was logged.

After making these initial changes to the materials, the ART met again to review the revised drafts and further refine the materials using a similar approach to the first round. including the post-meeting survey. Following this meeting, adaptations were finalized.

Throughout the adaptation process, research members (ET, RM) kept in contact with all ART members via email and phone calls. ART members were able to provide ongoing feedback, meet individually with one or more research members, and take pauses from the study. All meetings were held virtually due to the COVID-19 pandemic.

### Description of adaptations

An adapted version of the Reach, Effectiveness, Adoption, Implementation, and Maintenance (RE-AIM) enriched enhanced framework for the reporting adaptation and modifications to evidence-based interventions (FRAME) [2, 6] was used to systematically document changes and their impact on the delivery of the CDST. FRAME was designed to support the systematic documentation of adaptations to evidence-based interventions in health services and public health research studies. FRAME was developed using a combination of a literature review, focus group, and coding process and allows for the documentation of both the content and process of adaptations. The RE-AIM expanded FRAME includes additional concepts about adaptations informed by the broadly used Reach, Effectiveness, Adoption, Implementation, and Maintenance framework. Therefore, RE-AIM enriched FRAME includes a number of key categories including how the intervention was modified, which aspects of the intervention are impacted by the modification, and the reason for the adaptation (i.e., to increase reach, effectiveness, adoption, implementation, or maintenance). In addition to the existing RE-AIM enriched FRAME constructs, we added four new categories. We added size and scope based on recent work [27]. Size is defined as amount of total intervention time (i.e., patient contact minutes) impacted by the adaptation, while scope is defined as the total number of intervention sessions impacted, both represented as percentages. We also tracked whether an adaptation was made for a specific population (i.e., serious mental illness, Veterans/military, other). Finally, we tracked whether a change was considered in our initial list of suggestions but not implemented, and the reason for non-implementation. A matrix database was created based on the adapted RE-AIM enriched FRAME constructs to document adaptations and completed by two researcher ART members (ET, RM). They checked each other’s coding to ensure accuracy, with a third member (BR) as consensus expert. A non-ART research team member (ES) provided quality checking and summarized the data.

## Results

### Rapid matrix analysis results

The matrix analysis yielded 510 coded entries (example quotes in Table 2; full summary in Table 3). The most commonly discussed topics were Veteran experiences (40 entries), Veteran benefits of CDST (34 entries), the assertiveness skills training model (called “ASAP”) (34 entries), and the vignettes and roleplays (34 entries). 57% of entries were associated with a suggestion. Most suggestions were either to keep (120 entries) or adapt (107 entries) a component. These suggestions varied in level of specificity; specific suggestion included moving a particular bullet point or revising a vignette; while less specific suggestions described possible issues, like ruptures in the therapeutic relationship, without describing a specific solution.

**Table 2:** Example quotes from rapid matrix analysis describing initial response to CDST and suggested adaptations

Suggestions		Fit for Veterans and VA PRRC Setting	
Specific suggestions	“…summarize the differences between these four [communication] styles. I think very visually so I’m envisioning a four by four, like a cube, and each box: one is assertive, one is aggressive, one is passive aggressive, and then maybe a couple bullets under each one.” (clinician)	**Positive overall response**	“My overall impression is this could be really helpful. I’m all excited, I want to go through the groups if it comes to [PRRC].” (Veteran)
Non-specific suggestions	“In one of the role-plays in the provider’s manual it said the client can ask to talk to someone else if the provider will not communicate effectively with them. But I think, in the end, what happens when communication breaks down with a provider? How do you go about politely and professionally asking for a different provider? And at what point do you say it breaks down? Because you can’t always be asking for new providers.” (Veteran)	**Importance of empowerment and decisional autonomy for Veterans**	“When you’re in service and you’re not able to express your own opinions and thoughts or what have you. Being a part of the decision-making is really a step up to express that and to [have my treatment needs] heard is huge for Veterans.” (Veteran)
Barriers to Veteran engagement in CDM and CDST	Veteran: In my mind it sounds stupid. I need [my providers’] advice but I’m just nervous on asking for it. I feel like I should already know these answers Interviewer: And does that mean that you don’t tell them as much as you would otherwise? Veteran: Correct. Yeah, I tend to downplay a lot “Just not set people up for disappointment too, because if we’re going to relay to them ‘if you do this stuff your provider will do everything you want’ but that’s not realistic necessarily. It reminded me of [Social Skills Training] because it comes up a lot where Veterans will say ‘well I tried everything we covered in here and it still didn’t work’ and then they get sort of deflated.” (clinician)	**Communication norm mismatch between military and civilian contexts**	“There have been times, certainly for myself, where- I don’t even think [I was] just accused, I think it’s an accurate assessment that I was being aggressive with people, but I didn’t understand at the time I was being aggressive. Especially shortly after I got out of the military.” (Veteran)
Overcoming psychological barriers to Veteran engagement in CDM and CDST	“Let [the Veterans] know it’s okay, that you’re not going to be shunned away from just because there’s something that [clinicians] don’t agree with.” (Veteran) “Before you go to talk to a provider, take a moment to calm yourself. And after these interactions, if you’re feeling frustrated, do something to soothe yourself or calm yourself.” (Veteran)	**Importance of easy to deliver group materials for VA PRRC clinicians**	“It’s a heavy cognitive load for me anyway- running a group as you’re trying to maintain engagement, trying to deliver content, trying to the stick to the protocol, trying to rein people in who are (laughs) getting a little off- so if the manual is easy to use and clear as possible it just makes it less stressful for me to facilitate.” (clinician)

**Table 3:** Summary of matrix analysis, including stakeholder group and whether each code was associated with a suggestion to adapt CDST

	**Veteran**	**Clinician**	**Keep**	**Adapt**	**Add**	**Remove**	**No Suggestion**	**Total**
**Veteran experiences**	40	0	3	0	0	0	37	40
**ASAP model**	23	11	9	15	2	0	8	34
**Veteran benefit**	33	1	6	1	2	0	25	34
**Vignettes and role-plays**	17	17	11	6	12	1	4	34
**Intervention structure**	17	10	6	8	4	0	9	27
**Veteran handbook**	24	1	6	12	2	0	5	25
**Collaborative decision-making concept**	21	3	0	2	0	0	22	24
**Treatment teams psychoeducation and worksheet**	16	8	11	4	4	0	5	24
**Complaints, provider disagreement and conflict**	13	10	4	8	3	0	8	23
**SCALIE model**	17	6	7	7	0	0	9	23
**Collaborative decision-making psychoeducation**	15	7	11	3	3	0	5	22
**NOW model**	16	6	12	4	0	0	6	22
**Homework**	13	8	4	0	8	0	9	21
**Appropriateness and adaptation needs for Veteran population**	13	6	5	6	1	0	7	19
**Overview card**	16	3	6	1	2	0	10	19
**Veteran ease of use**	10	9	2	9	5	0	3	19
**Clinician manual**	2	15	3	6	3	0	5	17
**Combined collaborative decision-making hand-out**	11	6	4	8	0	0	5	17
**Virtual delivery considerations**	12	5	0	1	1	0	15	17
**Appropriateness and adaptation needs for CORE clinical setting**	6	10	2	3	2	1	8	16
**Advocacy discussion and roleplay**	4	7	4	0	2	0	5	11
**Service delivery manual**	2	8	1	0	6	0	3	10
**Clinician ease of use**	0	9	3	3	1	0	2	9
**Clinician experiences**	0	3	0	0	0	0	3	3
**Total**	341	169	120	107	63	2	218	510

Additionally, both Veterans and clinicians reported concerns about potential barriers, as well as ways to overcome them. These discussions were usually coded as suggestions to identify mechanisms within CDST to support Veterans to overcome barriers and increase the effectiveness of CDST. A common barrier for Veterans was potential psychological and interpersonal barriers that could prevent use of skills taught in CDST, even for Veterans who found those skills valuable. For example, one Veteran reported that using the skills would be *“scary”* but it was possible to *“conquer that fear.”* Multiple Veterans offered suggestions to help overcome fear and nervousness to use the skills, mostly focused on validating Veterans’ emotions, empowering them, and helping them manage difficult emotions and interactions.

Clinicians indicated some concern that aspects of CDM and associated psychoeducation might be misinterpreted, leading Veterans to believe that using CDM skills should always result in providers agreeing with Veteran preference for care. The clinician suggested discussing how to *“define success”* when using the targeted skills. Although the preferred outcome may not be achieved, using the skills makes that more likely, and has other benefits including speaking up for yourself and developing self-respect. Similarly, one Veteran commented, *“I did like that [the CDST materials] validated and acknowledged that sometimes treatment providers won’t be cooperative.”* These more open-ended suggestions were noted in the analysis for further discussion at the ART meeting to ensure that they were addressed effectively.

In terms of fit for Veterans and the VA PRRC setting, both clinicians and Veterans reported it was a generally good fit as is, without requiring *“a lot of modifications,”* according to one clinician. Suggestions related to improving fit for the setting were fairly minor, including changing language to fit the terms used in the setting; and adding an examples about romantic relationships and military sexual trauma because these are frequently a priority for Veterans in the setting. Additionally, both Veterans and clinicians made recommendations to increase usability for Veterans with significant cognitive impairments due to traumatic brain injury, including more visual aids and less dense text throughout the manual.

Of the comments that were not suggestions, many related to overall response to CDST, the collaborative decision-making concept, and its fit and appropriateness for the population and setting. Veterans and clinicians alike had an overall positive response to collaborative decision-making as a concept, and to CDST specifically.

Other key themes included aspects of the intervention that were particularly important for Veterans. Multiple Veterans reported that military service members typically have little autonomy over decision-making, and little ability to express emotions, making it more important empowering to learn and use those skills. Additionally, multiple Veterans reported that communication styles between the military and civilians vary, making learning assertiveness skills particularly important for Veterans who have frequently been trained to use a more aggressive style during military service. These Veterans noted that this gap in communication norms had led to negative reactions from clinicians, decreasing ability to participate in CDM.

Potential barriers for clinicians delivering CDST included ease of delivery. Although overall clinicians found the manual to be *“organized”* and containing all the material they would need to start delivering CDST, increasing visual aids and in-text reminders for clinicians would be helpful for situations where clinicians were delivering it *“on the fly,”* rather than being able to *“study”* beforehand.

With respect to implementation feasibility, clinicians largely commented that CDST in its current form is structurally similar to the other groups currently delivered in the service setting, including in terms of the number of sessions, length of sessions, and other aspects of its structure and emphasis on skill development. Clinicians recommended that the service delivery manual include recommendations for dealing with intermittent attendance and infrequent homework completion, given that these are common concerns in the setting.

### Iterative adaptations and post meeting surveys

The first ART Meeting included two researchers, two clinicians, and three Veterans. Twelve major topics were discussed including increasing ease of use and delivery; how mood and symptoms may impact ability to use CDM; supporting Veterans who feel uncomfortable or cautious using CDM; and specific changes to handouts, examples, and other materials. Early drafts of adaptations were presented during topics when relevant.

The post meeting survey was updated based on the major adaptations discussed during the meeting and specific adaptations proposed. All of the Veterans and clinicians present completed the post-meeting survey. Most ART members reported that they felt “completely” heard and understood during the meeting; one person reported feeling “somewhat” heard and understood. ART members rated most adaptations as important and beneficial to Veterans; no adaptations were rated as unimportant or harmful. Responses to the open-ended item about most important topics discussed included increasing ease of use for both Veterans and clinicians (*n* = 2 ART members), the role plays and examples (*n* = 2), assertiveness (*n* = 1), and managing emotional dysregulation (*n* = 1).

Ratings related to ease of engagement and ease of delivery were mixed, with most adaptations rated a range from *“very easy”* to *“somewhat difficult.”* The exception was one worksheet, which was expanded to include a completed example without increasing the amount of time dedicated to teaching the worksheet; this was rated *“very difficult”* to deliver by one ART member.

The second ART meeting included two researchers, two clinicians, and one Veteran. One Veteran met with the research team separately due to scheduling constraints. Twelve major topics were discussed including increasing ease of use and delivery through methods including new and adapted visual aids and tables; enhanced reminders for clinicians to use evidence-based learning strategies for group and individual interventions; changes to specific worksheets; addition of coping skills sections and card; and expanding handling conflict and disagreements section. Drafts of adaptations were presented.

The post-meeting survey mirrored the structure of the first but differed in content based on what was discussed during the meetings. The survey included images of drafted changes that were proposed when appropriate (e.g., adapted worksheets). Both Veterans and one clinician completed the survey. All reported that they felt “completely” heard and understood. Similar to the first survey, all topics were rated as important and beneficial to Veterans. In the open-ended questions, ART members reported that *“all”* the topics were important (*n* = 2), changes to handouts and examples were most important (*n* = 1), and talking to a provider was most important (*n* = 1). Unlike the first survey, ratings of ease of engagement and ease of delivery ranged between *“very easy”* and *“somewhat easy”* for nearly every topic. The exception was a suggested addition to a worksheet, which had one rating of *“somewhat difficult”* to engage in.

### Characterizing final adaptations in FRAME

There were 164 adaptations entered into the modified FRAME, which included six adaptations that were ultimately not incorporated into CDST for reasons including compromising research integrity and redundancy with superior adaptations. Therefore, a total of 158 adaptations were completed. Please see Table 4 for a summary of FRAME and Table 5 for examples of adaptations made.

**Table 4:** Summary of adaptations made using the Reach, Effectiveness, Adoption, Implementation, and Maintenance (RE-AIM) enriched, expanded Framework for Reporting Adaptations and Modifications (FRAME)

	Initiator of adaptation															
**Adaptation constructs**	**Research staff**	**Veteran**			**Clinician**		**Clinician & Veteran**		**Clinician & Research staff**		**Veteran & Research staff**		**Clinician, Research staff & Veteran**			**Total**
Which of the following was the primary type of change(s) involved?																
**Adding a component**	27	13			27		7		3		1		0			78
**Extending a component**	4	6			17		3		1		0		1			32
**Tailoring to individuals**	2	9			7		3		0		0		0			21
**Substituting for a component**	5	5			8		1		0		0		0			19
**Otherwise changing the intervention**	0	1			6		1		1		0		0			9
**Changing the order of components**	0	2			1		0		0		0		1			4
**Other change**	0	2			1		1		0		0		0			4
**Loosening the structure or protocol**	1	0			1		0		0		0		0			2
**Removing a component**	1	0			0		0		0		0		0			1
**Condensing a component**	0	0			0		0		0		0		0			0
**Integrating with other programs we are doing**	0	0			0		0		0		0		0			0
**Repeating a component**	0	0			0		0		0		0		0			0
What was changed- component of CDST																
**Service delivery manual**	16	7			20		1		0		0		0			44
**Psychoeducation**	2	3			5		3		1		0		0			14
**Worksheets**	0	3			4		3		2		0		1			13
**Examples**	2	0			7		4		0		0		0			13
**Protocol**	13	0			0		0		0		0		0			13
**At-home practice**	3	1			6		0		0		0		1			11
**Managing conflict and disagreements**	1	8			2		0		0		0		0			11
**Other component**	2	0			6		2		0		0		0			10
**Assertiveness skills (including ASAP)**	0	1			8		0		0		0		0			9
**Treatment teams**	0	2			3		0		0		0		0			5
**Clinician instructions**	0	3			1		0		1		0		0			5
**Goal identification and planning skills (including NOW)**	0	1			3		0		0		0		0			4
**Problem solving skills (including SCALIE)**	0	1			3		0		0		0		0			4
**CDST basics card**	0	1			0		3		0		0		0			4
**Coping skills**	0	2			0		1		0		0		0			3
**Role-plays and other in-session practice**	0	0			2		0		0		0		0			2
**Advocacy**	0	0			2		0		0		0		0			2
**Practice group**	1	0			1		0		0		0		0			2
**Active engagement of participants**	0	2			0		0		0		0		0			2
**Empowerment**	0	1			0		0		0		0		0			1
**Discussion**	0	1			0		0		0		0		0			1
**Coping skill card**	0	0			0		0		0		1		0			1
**Other active engagement of participants**	0	0			0		0		0		0		0			0
What was the basis for the adaptation																
**Based on our knowledge or experience of working with patients ("knowing the Veterans we serve, I know we'll need to consider XX")**	15	5			36		10		2		1		1			70
**Based on patient knowledge or experiences ("Based on my past experiences, I know I would struggle with this because…")**	3	29			10		12		2		0		2			58
**Based on a framework (for example collaborative decision-making [CDM] principles)**	17	5			16		4		2		1		0			45
**Other**	0	1			10		0		0		0		0			11
**Based on our vision or values (VA's vision, interviewee's values, etc.- ex: "VA promotes Veteran centered care…" or "I don't think homework is important…")**	4	1			3		1		0		0		0			9
**Based on pragmatic/practical considerations (for example “this is the only way it would work” "our patient flow requires…")**	8	1			0		0		0		0		0			9
**Based on QI data, summary information or results (during implementation, identifying patterns in delivery indicating need for changes to improve attendance or at home practice completion)**	0	0			0		0		0		0		0			0
**Based on financial incentives/payment**	0	0			0		0		0		0		0			0
Why was the change made																
**To enhance the impact or success of the intervention for all or important subgroups (effectiveness)**	28	34			48		14		3		1		1			129
**To make the intervention delivered more consistently; to better fit the PRRC, clinician needs, patient flow or EHR; for practical reasons (implementation)**	3	0			15		0		1		0		1			20
**Other- describe**	4	0			1		0		0		0		0			5
**To increase the number or type of patients contacted (reach)**	4	0			0		0		0		0		0			4
**To make it possible to involve more teams, team members or staff (adoption)**	1	1			0		0		0		0		0			2
**To institutionalize or sustain the intervention (maintenance)**	0	0			0		0		0		0		0			0
**To respond to external pressures or policy**	0	0			0		0		0		0		0			0
**To save money or other resources (implementation)**	0	0			0		0		0		0		0			0
Was this adaptation specifically to address population-specific needs?																
**No**	35	27			54		11		3		1		1			132
**Veteran/military**	2	4			7		2		1		0		1			17
**Serious mental illness**	2	7			5		2		0		0		1			17
**Other population**	1	0			1		0		0		0		0			2

**Table 5:** Examples of adaptations made to CDST

Original content	Adapted content
Skills training models appear as text, with tables and worksheets	When each model is discussed, its icon appears. For example, the following appears with the ASAP assertive communication model:
Discuss and validate past negative experiences with mental health clinicians that might impact current comfort engaging in collaborative decision-making	Add coping skill practice and coping skill card (front side below) to decrease anxiety and increase confidence
Range of examples and vignettes, but none targeted specifically for Veterans	Add new examples including Veteran-specific examples about disclosing military sexual trauma and seeking care outside of VA
Address standard components of evidence-based skills training and cognitive-behavioral interventions (e.g., agenda setting, homework review, session review) briefly in Clinician Manual and more thoroughly in the Service Delivery Manual and clinician training	Add specific outlines for agendas, homework review, and session review in each session; see for example, the session review for session 1 below:

Considering types of changes made, 78 adaptations added a component to CDST, and 32 extended an existing component. Only one component (jargon-based language) was removed. The size of most adaptations was small; impacting approximately 2% of less of the intervention. For example, one extended component increased psychoeducation about communication styles by adding a completed assertive communication table that compares elements of assertive, aggressive, passive, and passive-aggressive communication was estimated to take about five minutes of one session to describe (equaling 1% of total intervention time). The three largest adaptations (adding coping skills, adding new vignettes and roleplays, and formalizing agenda setting and homework review processes) were estimated to have a size of 10%-16%.

The scope of most adaptations was one session. Eleven adaptations impacted all sessions. However, size and scope were not necessarily aligned. For example, one adaptation tailored language to Veterans and VA, including changing “client” to “Veteran” in all materials. This change had a size of 0%, as it did not take any in-session time, but a scope of 100%, as it impacted every session.

Notably, the Service Delivery Manual was developed during the adaptation process and so all of its elements were categorized as added components (44 total adaptations). While these added to the overall materials and changed how a clinician would experience, and ideally deliver, CDST, they do not impact the length of the intervention itself and so were not included in size and scope calculations. The completed initial version of the Service Delivery Manual includes information about Veterans with SMI, including common symptoms, trauma, cognition, functioning, and military culture; methods of tailoring group therapy for SMI populations, including teaching methods for participants with cognitive or learning impairments; an overview about collaborative decision-making; specific recommendations for facilitating CDST, including ways to encourage at home practice completion, approved modifications, and troubleshooting common problems like intermittent attendance; and the full list of examples from the manual along with nine additional new examples that can be used to tailor a session to a given group.

All of the stakeholder groups contributed to the FRAME by providing initial ideas and suggestions. Regarding primary contributors of initial suggestions, clinicians contributed 54 adaptations, research staff contributed 35, and Veterans contributed 27. Additionally, 16 adaptations were contributed by a combination of stakeholder groups, usually through the conversations during ART meetings. In terms of CDST components, the components clinicians initiated the most adaptations were the Service Delivery Manual (*n* = 20 adaptations), assertiveness skills (*n* = 8), and examples (*n* = 7). Veterans initiated the most adaptations to the managing conflict and disagreement component (*n* = 8 adaptations), and the Service Delivery Manual (*n* = 7). Research staff initiated the most adaptations to the Service Delivery Manual (*n* = 16 adaptations).

Regarding the basis for each adaptation, the most common basis was *based on our knowledge or experience of working with patients ("knowing the Veterans we serve, I know we'll need to consider XX"),* with 70 total adaptations, and 36 of those initiated by clinicians. Another 58 adaptations were based on *patient knowledge or experiences ("Based on my past experiences, I know I would struggle with this because…")* with 29 of these initiated by Veterans and 12 initiated by a combination of Veterans and clinicians. The third most frequent basis was a *framework*, including CDM principles and cognitive behavioral therapy, which made up 45 adaptations. Nearly all (*n* = 129) of the adaptations were made to *enhance the impact or success of the intervention for all or important subgroups,* associated with the Effectiveness dimension of RE-AIM. The second most common category was *making the intervention delivered more consistently; to better fit the PRRC, clinician needs, patient flow or EHR; for practical reasons;* the Implementation dimension of RE-AIM, with 20 adaptations. A relatively small number of adaptations (*n* = 36) were completed specifically to address population-specific needs. Of these, 17 were for the Veteran/military population, 17 were for the SMI population, one was for race and ethnic minority groups, and one was for people who have experienced trauma.

## Discussion

This iterative, mixed-methods, and stakeholder-engaged adaptation of CDST was aided by the IM-Adapt model and the RE-AIM enriched FRAME, which increased ability to effectively complete and document adaptations and create a clinical product for immediate and scalable application. Engaging diverse groups of stakeholders through multiple methods enhanced the quality and the depth of data and final product. The ART process further developed rapport, and enhanced ability to deeply understand the needs, values, and priorities of diverse stakeholders. Evidence from the post ART meeting surveys indicated that Veteran and clinician ART members perceived the adaptations to be important and beneficial to Veterans. Further, the improvements in perceived ease of participation and delivery from the first ART meeting to the second indicated that adaptations improved perceived implementation feasibility. These data provide support that the adaptation was successful and likely to improve fit for Veterans and the VA clinical context, although a clear next step is to implement the adapted materials and test their effectiveness and feasibility in this context.

The iterative and group-based approach to adaptation allowed for the refinement of ideas over time, increasing ability to optimize adaptations to target nuanced issues. For example, one of the largest adaptations, the addition of coping skill psychoeducation and practice, began as a more abstract discussion of the potential barriers to using CDST. ART Veterans described how mood, symptoms, lack of confidence, and discomfort around using CDM might impact whether Veterans might choose to use CDM, and if they did, how effective they might be. Over the course of the adaptation process, the team decided to integrate coping skill practice, and made specific decisions about which coping skills to integrate and when to practice them during each session. Although these coping skills were new to CDST’s forms, the function of increasing comfort and confidence in initiating and engaging in CDM was an integral part of CDST’s initial conceptualization, making this significant adaptation a good fit. Additionally, the team debated over this addition because most of the coping skills added are already commonly taught in other individual and group interventions in this setting. However, the team decided that most Veterans would benefit from explicitly discussing and practicing how to apply these skills to CDM.

We tracked size and scope of the completed adaptations; most were small. However, the team found that these metrics, and particularly size, did not necessarily translate to perceived impact of a given adaptation. For example, the change from “client” to “Veteran” across all CDST materials is a relatively small change, but meaningful given how important Veteran identity is to many participants.

Overall, Veterans found the content particularly well-fitting for the Veteran population, both in terms of its purpose, to empower Veterans and increase Veteran voices in mental health care, and in terms of some of its specific functions, including assertiveness training and use of validation. This may be why there were relatively few adaptations made specifically to increase fit for Veterans. Most of the adaptations made were not tailored for any specific population, and therefore may increase CDST’s effectiveness and/or implementation feasibility across groups.

The ART broadly found that CDST fit well with the VA PRRC clinical context, increasing its implementation feasibility. Still, a priority of these adaptations was to increase ease of delivery for busy clinicians with strong underlying skill sets but who may need in-manual reminders to maintain fidelity and would benefit from recommendations about managing common group intervention concerns. Some of these adaptations, for example adding bullet points of key points at the end of each session to aid in session review, were formalizations of forms that were intended to be a part of CDST. This served as an important reminder about feasibility: that clinicians benefit from and appreciate support to implement evidence-based strategies, even those they are already well-trained in.

One concern sometimes described about engaging multiple groups of stakeholders in research, is that a more powerful group’s voice may intentionally or unintentionally silence a less powerful group. This was certainly something we considered and monitored through the study process, given that we included researchers, clinicians, and Veterans with psychosis in a single, interactive team. Overall, we believe we were successful in engaging multiple types of stakeholders, and that these interactions positively impacted the study, echoing other stakeholder-engaged adaptations in VA settings [1]. There were interactions during group meetings between Veterans and clinicians that helped identify and refine adaptations that would not have been achievable if these two groups were kept apart throughout the study. Formal feedback on the post-meeting surveys and informal feedback given to the research staff generally supports that stakeholders felt heard and able to participate to the extent they desired. The team noticed that clinicians often made sure that Veterans had time to speak during meetings, and checked in that their comments were reasonable and made sense to Veterans. Additionally, the three groups of stakeholders were fairly equally represented in terms of the adaptations that were initially suggested by them. Stakeholders of all groups were provided flexible options so that they could meet with research staff individually, skip meetings if needed/preferred, and make other requests so that their voices could be heard on their own terms. We also included all groups in the writing of this paper.

However, we fully acknowledge that this is a complex process, and success depends on the methods of the study, the researchers and stakeholders involved, and the environment created. It is essential that an engaging and non-confrontational atmosphere is facilitated by leaders of a multi-stakeholder team. Based on this study, we recommend open communication and elicitation of contact and participation preferences; flexibility around participation level and adaptability to changing needs throughout the study; and iterative contacts to encourage rapport development and disclosure as trust increases. We also encourage team leaders to implement the key tenets of participatory action research during individual and group meetings; this includes demythologizing research practices and expanding both the practice and the critique of research within the hands of all team members; centering community and community member values; developing an atmosphere where stakeholders are respected and heard; and intentionally displacing decision-making power from leadership and other default individuals and entities to balance equitably across all team members and groups [28–30]. In our study, this included encouraging open discussion and disagreement with team leadership and making adaptation decisions based on team consensus, with particular attention to Veteran preferences, rather than giving more weight to team leader preferences. It also meant maximizing accessibility to the entire research process including participation in products and compensating stakeholders for that participation. Regardless of whether stakeholders of different groups interact with each other, we strongly echo the ADAPT guidance to meaningfully include Veteran (and/or other relevant patient) stakeholders and clinician stakeholders in adaptation and implementation teams [31].

### Limitations

Adaptations are naturally driven by the local setting, participants, and other contextual factors. The methods used in this study and the resulting modified version of CDST may require further adaptation for effective use in other settings and with other populations. It is possible that other VA PRRCs might need to make adaptations to the resulting CDST materials for their own purposes. The Service Delivery Manual developed in this study is intended to serve as a document that will both inform future adaptations and increase ability to tailor CDST to other PRRC contexts, and to be updated as CDST experts learn more about how to effectively deliver and tailor CDST to these contexts. Post-implementation evaluation of the adapted CDST intervention is needed to identify its effectiveness and implementation feasibility for its target population and setting.

## Conclusions

In this study, a multi-stakeholder team of Veterans, VA clinicians, and researchers was assembled to iteratively and collaboratively adapt Collaborative Decision Skills Training (CDST) using a mixed methods approach. CDST was well-received in terms of its content and feasibility; however, 158 adaptations were completed. Most of these adaptations were small in size and scope and were intended to increase effectiveness. Veteran and clinician team members reported that these adaptations were important and beneficial to Veterans, indicating adaptation success. The next step will be to test the adoption, implementation, reach, effectiveness, and sustained use of the adapted CDST intervention in diverse VA settings.

## Data Availability

The datasets during and/or analyzed during the current study available from the corresponding author on reasonable request.
